# Impact of annulus-cusp mismatch on mid-term outcomes of aortic valve repair with valve-sparing aortic root replacement

**DOI:** 10.1093/icvts/ivaf048

**Published:** 2025-02-26

**Authors:** Go Yamashita, Atsushi Sugaya, Jiro Sakai, Shingo Hirao, Tatsuhiko Komiya

**Affiliations:** Department of Cardiovascular Surgery, Kurashiki Central Hospital, Kurashiki, Japan; Department of Cardiovascular Surgery, Kurashiki Central Hospital, Kurashiki, Japan; Department of Cardiovascular Surgery, Kurashiki Central Hospital, Kurashiki, Japan; Department of Cardiovascular Surgery, Kurashiki Central Hospital, Kurashiki, Japan; Department of Cardiovascular Surgery, Kurashiki Central Hospital, Kurashiki, Japan

**Keywords:** annulus-cusp mismatch, aortic valve regurgitation, aortic valve repair, coaptation length, valve-sparing aortic root replacement

## Abstract

**OBJECTIVES:**

This study aimed to investigate mid-term outcomes of aortic valve repair with valve-sparing aortic root replacement based on different grades of annulus-cusp mismatch and identify optimal aortic root geometries for this procedure.

**METHODS:**

A retrospective analysis was conducted between October 2011 and July 2022. Patients were stratified into three groups based on predicted coaptation length calculated using an annulus-cusp mismatch formula: no-mismatch (coaptation length > 4 mm, *n* = 52), mild-mismatch (2 mm ≤ coaptation length ≤ 4 mm, *n* = 28) and severe-mismatch (coaptation length < 2 mm, *n* = 25), and mid-term outcomes were compared.

**RESULTS:**

We included 105 patients who underwent valve-sparing root replacement using the reimplantation technique. During the median follow-up of 6.0 years, 21 moderate aortic valve regurgitation events and 6 reoperation events were observed. No significant inter-group differences in overall survival or cumulative incidence of cardiac death or hospitalization for heart failure were observed. However, the groups significantly differed in the cumulative incidence of moderate aortic regurgitation at 5 years (2.0%, 14.8% and 60.1% for no-mismatch, mild-mismatch and severe-mismatch groups, respectively; *P* < 0.001) and cumulative incidence of reoperation at 5 years (0%, 0% and 11.8%, respectively; *P* = 0.002).

**CONCLUSIONS:**

Our findings suggest that severe annulus-cusp mismatch is associated with higher rates of valve regurgitation and reoperation following aortic valve repair with valve-sparing aortic root replacement; however, larger studies are needed for confirmation. Preoperative computed tomography-based assessment of annulus-cusp mismatch shows promise in surgical planning and patient selection for aortic valve repair procedures.

**CLINICAL REGISTRATION NUMBER:**

4392

## GRAPHICAL ABSTRACT

**Figure:**
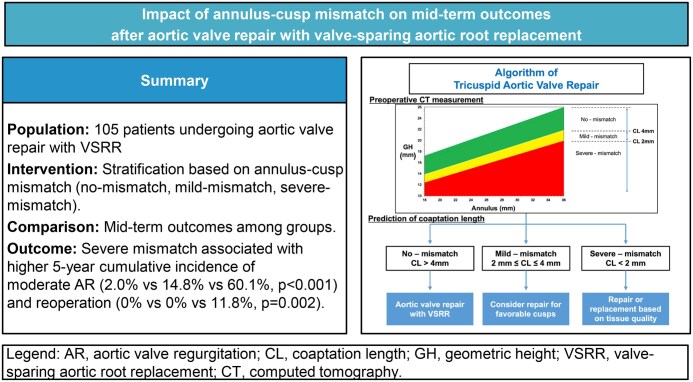

## INTRODUCTION

Aortic valve repair for aortic valve regurgitation (AR) has gained widespread acceptance as an established procedure. However, its long-term durability and difficulty in patient selection remain significant challenges due to the complex interplay between valve morphology and surgical technique.

Achieving adequate coaptation by ensuring sufficient cusp size to the aortic annulus diameter is essential for successful repair. Several techniques, including aortic valve reimplantation [1], suture annuloplasty [2] and external ring annuloplasty [3], have been developed to enhance coaptation, prevent aortic regurgitation recurrence and improve long-term durability by optimizing valve geometry. Our institution primarily employs the reimplantation technique [4] for valve-sparing aortic root replacement (VSRR) to address both annular stabilization and cusp coaptation.

Our previous study reported on the limitations of cusp size for aortic valve repair based on preoperative computed tomography (CT) scans, concluding that small cusp size is not a contraindication for aortic valve repair [5]. This finding has expanded the pool of potential candidates for this procedure. To further refine patient selection, we have since established a three-tiered grading system for annulus-cusp mismatch. However, the impact of varying degrees of mismatch on mid-term outcomes remains unclear.

Therefore, this study aimed to investigate the mid-term outcomes associated with different grades of annulus-cusp mismatch and identify optimal aortic root geometries for aortic valve repair with VSRR.

## MATERIALS AND METHODS

### Ethics statement

This retrospective, observational, single-centre study was approved by the Institutional Review Board of Kurashiki Central Hospital (Approval No.4392; Approval Date: 21 May 2024). The requirement for informed consent was waived due to the retrospective nature of the study.

### Study design

Preoperative CT measurements for aortic valve assessment were introduced at our institution in 2011. Between October 2011 and July 2022, 230 consecutive patients underwent aortic valve repair at our facility. After applying exclusion criteria (isolated aortic valve repair without VSRR, lack of preoperative CT imaging or images of poor quality, and unicuspid or bicuspid aortic valves), the final study cohort was established (Supplementary Fig. S1). Participants who underwent concomitant procedures, including coronary artery bypass grafting, other valve operations or thoracic aortic surgery, were included. Patient characteristics and operative data were obtained from medical records. Follow-up examination data, including survival and complications, were obtained from patient chart reviews at the outpatient clinic and telephone follow-up for patients who did not attend the outpatient clinic. Follow-up was completed in all patients.

### Preoperative CT measurements

**Figure 1:**
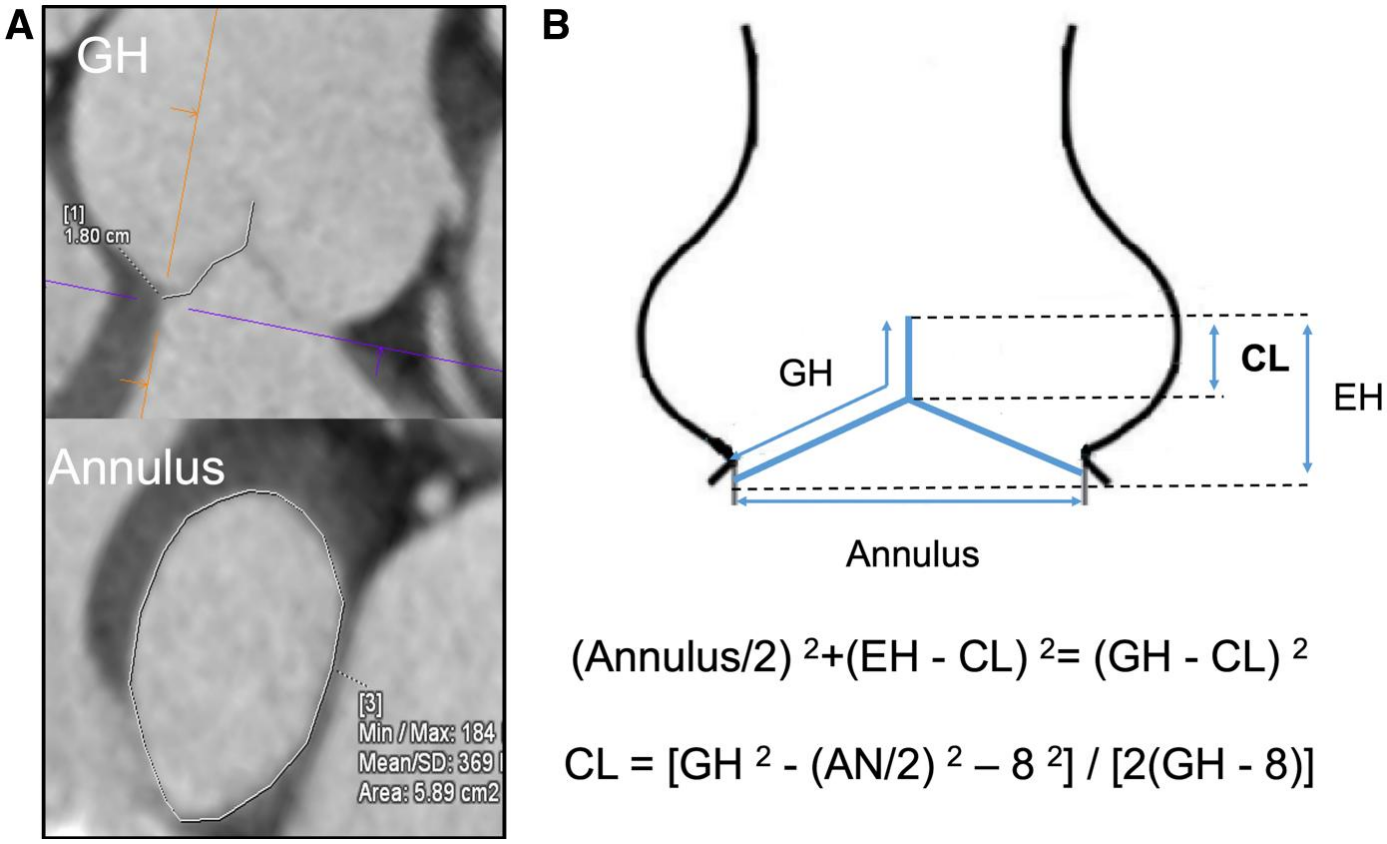
Measurement of the geometric height and basal annulus area on computed tomography (**A**). A simplified model of the aortic root and cusps. CL was derived using the Pythagorean theorem applied to the aortic root and cusp geometry (**B**)

The detailed methodology of CT measurements has been previously reported in our previous study [5]. Multidetector-row CT (MDCT) using a 128 2-slice dual-source scanner (Somatom Definition Flash, Siemens, Germany) was used to evaluate the aortic valve and aortic root. Image acquisition was synchronized with electrocardiography to facilitate retrospective analysis and reconstruction at decile intervals of the cardiac cycle’s RR interval. Data analysis was conducted using a specialized workstation (Syngo.Via, Siemens). End-diastolic images were primarily analyzed to assess cusp immobility, which is anticipated during this phase. Geometric height (GH) was quantified from the nadir of the sinus to the midpoint of the free margin (Fig. 1A). In instances of cusp prolapse, the deflected segment was delineated using an electronic stylus. The mean GH of the three cusps was incorporated into the analysis, and the basal annulus area was measured in a transverse plane intersecting the nadirs of the aortic valve. Each diameter was derived using the formula: area = πr^2^.

### Prediction of coaptation length and annulus-cusp mismatch stratification

Figure 1B illustrates the simplified model of the aortic root and cusp, consistent with previous reports. Coaptation length (CL), defined as the overlap length of the cusps at the centre, was calculated using a relational formula derived from the Pythagorean theorem. The effective height (EH) was preset at 8 mm for CL computation.


$${\left(\text{Annulus}/\text{2}\right)}^{\text{2}}+\;{\left(\text{EH }-\text{ CL}\right)}^{\text{2}}=\;{\left(\text{GH }-\text{ CL}\right)}^{\text{2}}$$

$$\text{CL }=\;\left[{\text{GH}}^{\text{2}}-\;{\left(\text{AN}/\text{2}\right)}^{\text{2}}–{\text{ 8}}^{\text{2}}\right]\;/\;\left[\text{2}\left(\text{GH }-\text{ 8}\right)\right]$$

**Figure 2:**
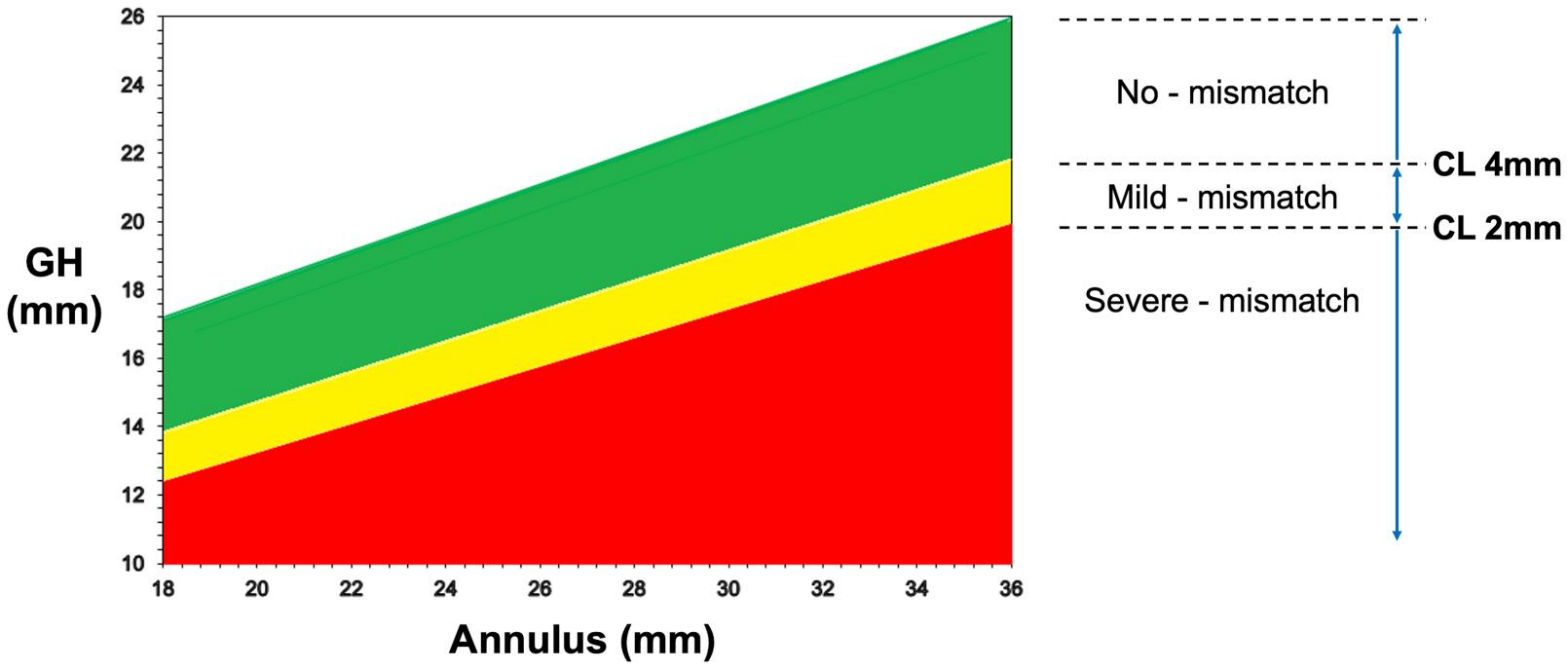
Stratification of annulus-cusp mismatch based on geometric height (GH) and annulus diameter. The graph illustrates the relationship between GH (*y*-axis) and annulus diameter (*x*-axis). Lines divide the graph into three regions: no-mismatch (CL > 4 mm, green), mild-mismatch (2 mm ≤ CL ≤ 4 mm, yellow) and severe-mismatch (CL < 2 mm, red)

Based on the predicted CL and annulus-cusp mismatch formula, patients were stratified into three groups: no-mismatch group (CL > 4 mm), mild-mismatch group (2 mm ≤ CL ≤ 4 mm) and severe-mismatch group (CL < 2 mm) (Fig. 2).

### Echocardiographic follow-up

All patients underwent transthoracic echocardiography within 2 weeks postoperatively and annually thereafter for follow-up.

### Surgical technique and preoperative planning

The surgical technique for aortic valve repair with VSRR has been previously described in detail [4]. Preoperatively, aortic valve geometry is assessed using three-dimensional high-resolution MDCT, including measurements of the annular size and aortic cusps. The predicted CL was then calculated using the annulus-cusp mismatch formula to determine the target annular diameter and Valsalva graft size. For instance, if the annular diameter is 30 mm and the mean GH is 17 mm, the predicted CL would be 0 mm. Setting a target post-repair CL of 4 mm (no-mismatch), the requisite annular diameter would be 24.7 mm, with a predetermined Valsalva graft size of 26 or 28 mm.

Intraoperatively, the preoperative measurements were verified and adjusted as needed. The VSRR technique was performed as described by David and Feindel [1]. A transverse incision in the ascending aorta facilitated the direct measurement of each aortic root component to corroborate CT findings [6]. Initial aortic valve repair was performed to optimize cusp margin length. Subsequently, the Gelweave Valsalva graft (Terumo; Tokyo, Japan) is secured to the basal annulus using first-row sutures, and flexible endoscopy assessed the post-repair valve morphology. Autologous pericardial patches were generally avoided due to their unfavourable long-term outcomes [7–10]. However, in cases of extensive fenestration, autologous pericardial patches may be employed. The surgical result was intraoperatively evaluated via transoesophageal echocardiography, where mild to no residual AR was considered satisfactory (if central and not eccentric).

### Statistical analysis

Continuous variables were presented as means ± standard deviation or medians (interquartile range [IQR]) depending on normality, which was assessed using the Shapiro–Wilk test. For comparison of continuous variables among three groups, one-way analysis of variance was used for normally distributed data, whereas the Kruskal–Wallis test was utilized for non-normally distributed data. The Jonckheere–Terpstra test assessed trends across ordered groups. Categorical variables were presented as counts and percentages and compared using Fisher’s exact test. Follow-up duration was calculated using the inverse Kaplan–Meier method, with death treated as censoring and actual follow-up time as an event, to avoid underestimating the follow-up period. The prespecified primary end-points were cumulative incidence of moderate AR and reoperation. Secondary end-points included overall survival, cumulative incidence of cardiac death and hospitalization for heart failure. Overall survival was estimated using Kaplan–Meier analysis. For cardiac death, the cumulative incidence function was estimated with non-cardiac death as a competing risk. For other postoperative outcomes (hospitalization for heart failure, moderate AR and reoperation), cumulative incidence functions were estimated considering death as a competing risk. Differences between groups were compared using Gray’s test. No adjustment for multiple comparisons was performed for the secondary end-points analysis. All statistical analyses were performed using EZR (Saitama Medical Center, Jichi Medical University, Saitama, Japan), a graphical user interface for R (The R Foundation for Statistical Computing, Vienna, Austria) [11], and statistical significance was set at a two-sided *P* < 0.05.

## RESULTS

The final study cohort comprised 105 patients who underwent aortic valve repair with VSRR using the reimplantation technique. These patients were stratified into three groups: no-mismatch (*n* = 52), mild-mismatch (*n* = 28) and severe-mismatch (*n* = 25). The overall median age ranged from 56.5 to 64.0 years, with a predominance of male patients (82.1–96.0%). Body mass index, body surface area and comorbidities were comparable among groups. Although preoperative echocardiographic parameters showed similar left ventricular end-diastolic dimensions and ejection fractions among groups, the left ventricular internal dimension in systole differed significantly (*P* = 0.045). Severe AR (type I and combined types I and II) was the most common presentation among the patients, with a trend towards higher prevalence in the severe-mismatch group (84.0%) compared with the no-mismatch (61.5%) and mild-mismatch (78.6%) groups (*P* = 0.082) (Table 1).

**Table 1:** Patient characteristics

Variable	No-mismatch	Mild-mismatch	Severe-mismatch	*P*-value
	*N* = 52	*N* = 28	*N* = 25	
Age, years (IQR)	64.0 (45.5–71.0)	56.5 (48.8–67.5)	61.0 (57.0–69.0)	0.523
Sex, male	45 (86.5)	23 (82.1)	24 (96.0)	0.318
Body mass index, kg/m^2^	23.3 ± 3.9	22.5 ± 3.5	23.7 ± 2.9	0.465
Body surface area, m²	1.77 ± 0.17	1.72 ± 0.15	1.74 ± 0.12	0.334
Hypertension	43 (82.7)	22 (78.6)	21 (84.0)	0.894
Hyperlipidemia	11 (21.2)	6 (21.4)	5 (20.0)	>0.99
Smoking	32 (61.5)	13 (46.4)	12 (48.0)	0.337
Diabetes	0	1 (3.6)	1 (4.0)	0.252
eGFR<45, ml/min/1.73 m^2^)	5 (9.6)	4 (14.3)	3 (12.0)	0.790
COPD	3 (5.8)	1 (3.6)	0	0.806
Previous stroke	4 (7.7)	3 (10.7)	1 (4.0)	0.802
Atrial fibrillation	5 (9.6)	2 (7.1)	2 (8.0)	>0.99
Resternotomy	1 (1.9)	3 (10.7)	1 (4.0)	0.185
Marfan	8 (15.4)	3 (10.7)	0	0.101
Preoperative transthoracic echo parameters				
LAD, mm	39.0 (32.0–45.3)	44.5 (35.0–46.3)	42.0 (38.0–45.0)	0.256
LVDd, mm	55.5 (49.5–63.3)	60.0 (56.5–64.0)	58.0 (55.0–63.0)	0.090
LVDs, mm	37.0 (32.0–44.3	41.0 (35.0–49.0)	43.0 (37.0–49.0)	0.045
LVEF, %	59.0 (54.8–60.3)	56.0 (52.0–60.0]	59.0 (51.0–60.0)	0.682
Preoperative AR				
None or trivial	5 (9.6)	2 (7.1)	0	0.348
Mild	5 (9.6)	0	0	0.074
Moderate	10 (19.2)	4 (14.3)	4 (16.0)	0.943
Severe	32 (61.5)	22 (78.6)	21 (84.0)	0.082
Classification of AR				
Type I	32 (61.5)	4 (14.3)	5 (20.0)	<0.001
Type II	2 (3.8)	7 (25.0)	7 (28.0)	0.002
Types I and II	16 (30.8)	14 (50.0)	12 (48.0)	0.156
Types I and III	0	0	1 (4.0)	0.238
Types II and III	0	1 (3.6)	0	0.505

Values are presented as *n* (%), means ± standard deviation, or medians (IQR).

eGFR: estimated glomerular filtration rate; COPD: chronic obstructive pulmonary disease; LAD: left atrial dimension; LVDd: left ventricular end-diastolic dimension; LVDs: left ventricular internal dimension in systole; LVEF: left ventricular ejection fraction; AR: aortic valve regurgitation.

### CT data and CL

Preoperative CT measurements revealed significant differences in GH across all cusps among the groups (*P* < 0.001 for all). The mean GH showed a significantly progressive decline in the no-mismatch (19.1 ± 1.4 mm) and mild-mismatch groups (17.6 ± 0.9 mm), which was further exacerbated in the severe-mismatch group (16.2 ± 1.1 mm) (*P* < 0.001 for trend). Conversely, the annulus diameter demonstrated significant differences with an increasing trend from the no-mismatch (26.4 ± 2.5 mm) to the severe-mismatch groups (27.8 ± 1.9 mm) (*P* = 0.030 for difference, *P* = 0.003 for trend). The predicted CL, calculated using the annulus-cusp mismatch formula, demonstrated significant differences with a declining trend in the no-mismatch (5.6 ± 1.3 mm) and mild-mismatch groups (3.1 ± 0.6 mm), which was further exacerbated in the severe-mismatch group (0.4 ± 1.4 mm) (*P* < 0.001 for difference, *P* < 0.001 for trend) (Table 2).

**Table 2:** Computed tomography data and coaptation length

Variable	No-mismatch	Mild-mismatch	Severe-mismatch	*P*-value	*P* for trend
	*N* = 52	*N* = 28	*N* = 25		
GH of RCC, mm	18.2 ± 1.6	16.6 ± 1.1	15.9 ± 1.6	<0.001	<0.001
GH of LCC, mm	18.8 ± 1.7	17.5 ± 1.4	16.2 ± 1.2	<0.001	<0.001
GH of NCC, mm	20.2 ± 1.8	18.6 ± 1.6	16.7 ± 1.4	<0.001	<0.001
Mean GH, mm	19.1 ± 1.4	17.6 ± 0.9	16.2 ± 1.1	<0.001	<0.001
Annulus, mm	26.4 ± 2.5	27.3 ± 2.0	27.8 ± 1.9	0.030	0.003
Predicted CL	5.6 ± 1.3	3.1 ± 0.6	0.4 ± 1.4	<0.001	<0.001

Values are presented as *n* (%), means ± standard deviation, or medians (IQR).

RCC: right coronary cusp; LCC: left coronary cusp; NCC: non-coronary cusp.

### Perioperative outcomes

Over the perioperative period, outcomes were evaluated for the no-mismatch, mild-mismatch and severe-mismatch groups, hereafter referred to in that order. Concomitant procedures were performed in 53.8%, 35.7% and 40.0% of patients, respectively (*P* = 0.241). The median Valsalva graft size differed significantly among groups (*P* < 0.001), with sizes of 28.0 mm (26.0–28.5), 26.0 mm (24.0–26.5) and 26.0 mm (26.0–26.0), respectively. Mean cardiopulmonary bypass time (*P* = 0.153) and cross-clamp time (*P* = 0.125) were similar among groups. Second aortic clamping due to AR was required in 7.7%, 17.9% and 16.0% of cases (*P* = 0.342). At discharge, none or trivial AR was observed in 96.2%, 89.3% and 80.0% (*P* = 0.065), respectively. No patients in any group had moderate or severe AR at discharge. In-hospital mortality was not reported in any group (Table 3).

**Table 3:** Intraoperative variables and short-term outcomes

Variable	No-mismatch	Mild-mismatch	Severe-mismatch	*P*-value
	*N* = 52	*N* = 28	*N* = 25	
Concomitant procedures	28 (53.8)	10 (35.7)	10 (40.0)	0.241
CABG	3 (5.8)	0	1 (4.0)	0.676
MVP	8 (15.4)	4 (14.3)	3 (12.0)	>0.99
TAP	0	2 (7.1)	0	0.124
MAZE	5 (9.6)	2 (7.1)	2 (8.0)	>0.99
VSD repair	1 (1.9)	0	1 (4.0)	0.490
PVR	1 (1.9)	0	0	>0.99
Hemiarch replacement	5 (9.6)	3 (10.7)	3 (12.0)	0.920
Partial or total arch replacement	9 (17.3)	1 (3.6)	2 (8.0)	0.202
Using pericardial patch	3 (5.8)	4 (14.3)	1 (4.0)	0.384
Valsalva graft size, mm	28.0 (26.0–28.5)	26.0 (24.0–26.5)	26.0 (26.0–26.0)	<0.001
24	4 (7.7)	8 (28.6)	4 (16.0)	0.049
26	11 (21.2)	13 (46.4)	17 (68.0)	<0.001
28	24 (46.2)	5 (17.9)	2 (8.0)	<0.001
30	13 (25.0)	2 (7.1)	2 (8.0)	0.064
Mean CPB time, min	240 ± 51.5	265 ± 68.8	245 ± 49.9	0.153
Mean cross-clamp time, min	188 ± 43.0	209 ± 48.8	194 ± 37.1	0.125
Second aortic clamping due to AR	4 (7.7)	5 (17.9)	4 (16.0)	0.342
None or trivial AR at discharge	50 (96.2)	25 (89.3)	20 (80.0)	0.065
In-hospital mortality	0	0	0	^†^

Values are presented as *n* (%) or means ± standard deviation.

† No in-hospital mortality was observed in any group; thus, statistical comparison was not applicable.

CABG: coronary artery bypass grafting; CPB: cardiopulmonary bypass; MVP: mitral valve plasty; TAP: tricuspid annuloplasty; VSD: ventricular septal defect; PVR: pulmonary valve replacement.

### Mid-term outcomes

**Figure 3:**
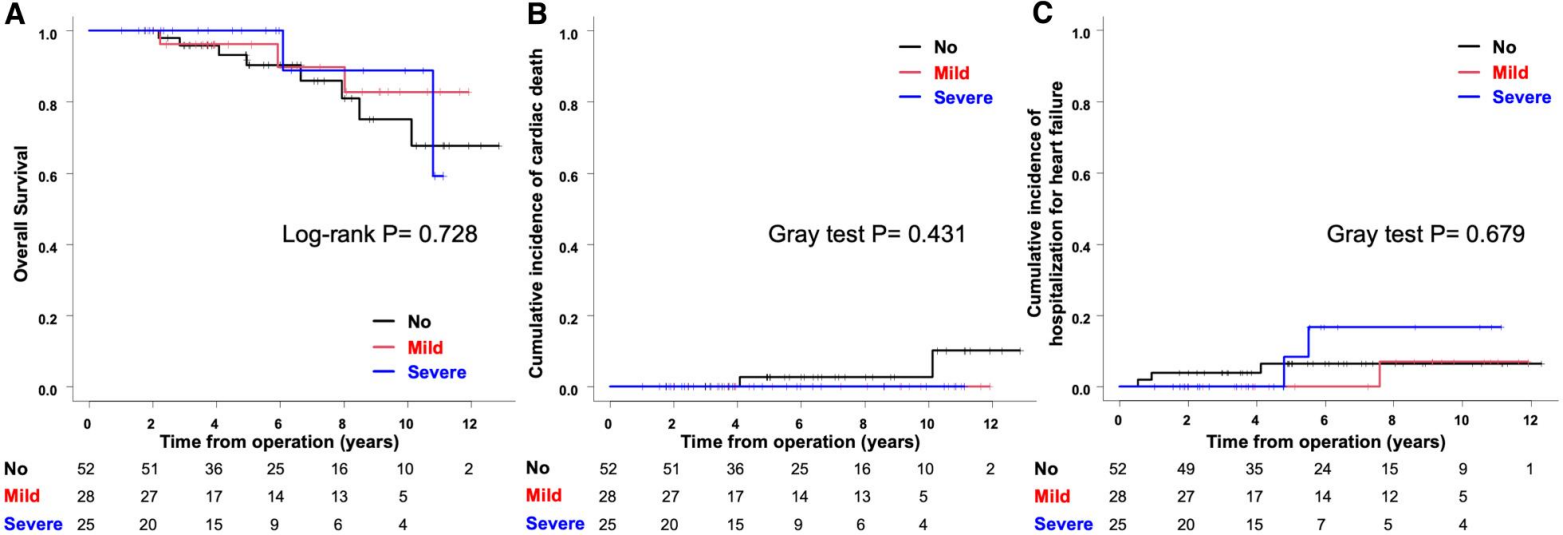
Overall survival (**A**). Cumulative incidence of cardiac death (**B**). Cumulative incidence of hospitalization for heart failure (**C**)

Over the median follow-up period of 6.0 years (IQR: 4.9–7.2 years, maximum: 12.9 years), we observed 13 deaths, 2 cardiac deaths, 6 hospitalizations for heart failure, 21 moderate AR events and 6 reoperation events. Long-term outcomes were evaluated for the no-mismatch, mild-mismatch and severe-mismatch groups, hereafter referred to in that order. Secondary end-points analysis showed no significant differences in overall survival among the three groups (log-rank *P* = 0.728; Fig. 3A). Overall survival rates at 5 and 10 years were 90.3%, 96.2% and 100%, and 75.2%, 82.8% and 88.9%, respectively. For the cumulative incidence of cardiac death, no significant differences were found (Gray test *P* = 0.431; Fig. 3B), with rates of 2.7%, 0% and 0% at 5 and 10 years across all groups. Similarly, analysis of cumulative incidence of hospitalizations for heart failure at 5 and 10 years did not show significant differences (Gray test *P* = 0.679; Fig. 3C), with rates of 6.5%, 0% and 8.3%, and 6.5%, 6.9% and 16.7%, respectively.

**Figure 4:**
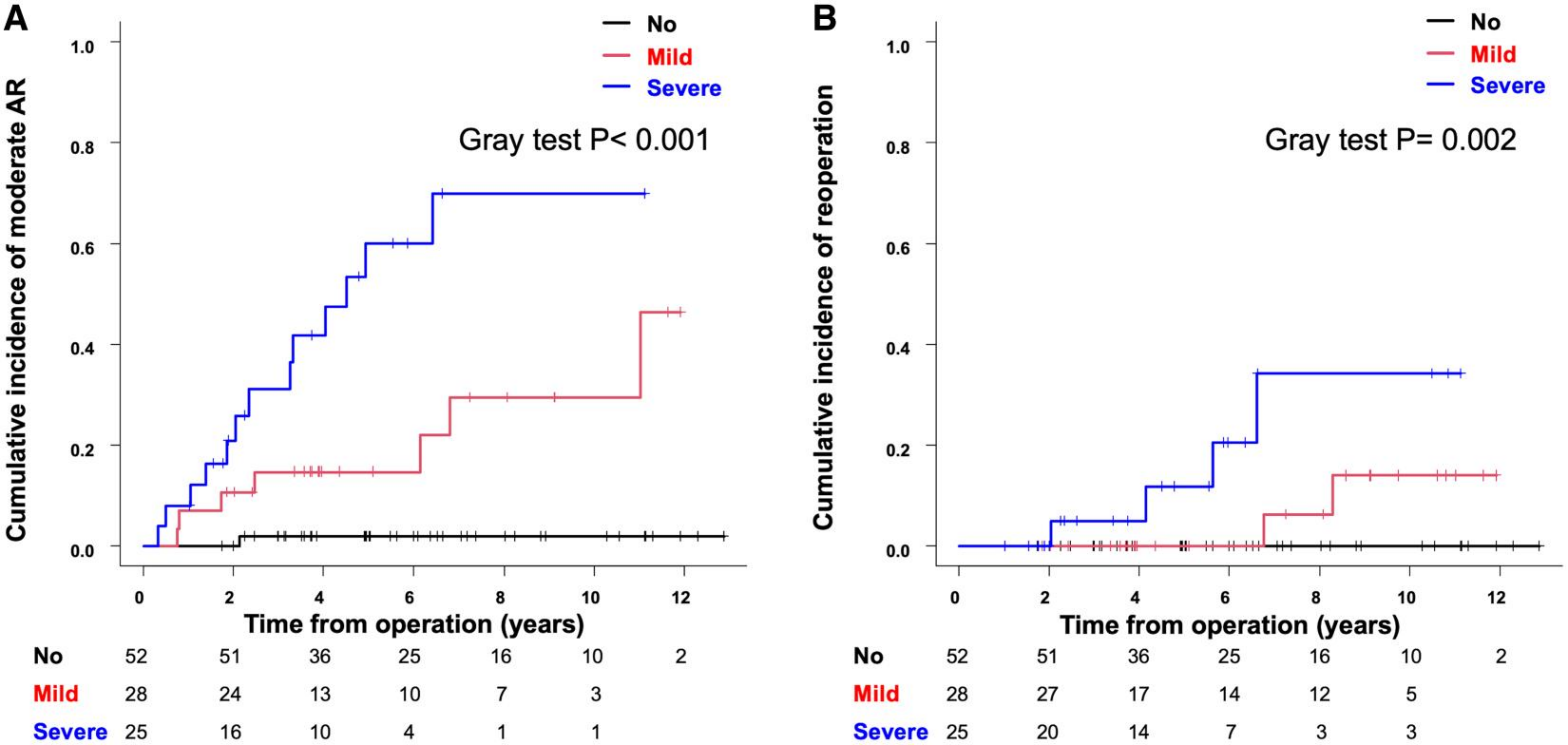
Cumulative incidence of moderate aortic valve regurgitation (AR) (**A**). Cumulative incidence of reoperation (**B**)

Primary end-points analysis revealed that the cumulative incidence of moderate AR differed significantly among the groups (Gray test *P* < 0.001; Fig. 4A). Cumulative incidence rates of moderate AR at 5 and 10 years were 2.0%, 14.8% and 60.1%, and 2.0%, 29.6% and 70.1%, respectively. Similarly, the cumulative incidence of reoperation differed significantly among groups (Gray test *P* = 0.002; Fig. 4B), with rates of 0%, 0% and 11.8%, and 0%, 14.0% and 34.2%, at 5 and 10 years post-surgery, respectively.

## DISCUSSION

This study highlights the significant impact of annulus-cusp mismatch on mid-term outcomes following aortic valve repair with VSRR. Severe annulus-cusp mismatch was associated with significantly poorer valve-related outcomes. Moreover, patients with severe mismatch exhibited higher cumulative incidence of moderate AR and lower rates of reoperation at 5 and 10 years post-surgery, compared with patients with no and mild mismatch. These findings suggest that the degree of annulus-cusp mismatch may be crucial in determining the long-term durability of aortic valve repair. Our classification system of annulus-cusp mismatch based on predicted CL provides a practical framework for preoperative assessment and patient selection for aortic valve repair procedures.

Our findings highlight the importance of achieving adequate coaptation during aortic valve repair. The severe-mismatch group, characterized by a predicted CL < 2 mm, showed a significantly higher rate of AR recurrence and reoperation, aligning with previous studies on the key role of coaptation in maintaining valve competence [6, 9, 12]. Based on our classification using 4 mm as the threshold for no mismatch, we observed favourable outcomes extending beyond 10 years on long-term follow-up in cases without mismatch (Supplementary Fig. S2). A prominent method for rectifying mismatch is patch augmentation, which was utilized in eight patients in our study due to large fenestrations (*n* = 7) and insufficient GH (*n* = 1). Although autologous pericardial patches have been associated with an increased risk of reoperation and recurrent regurgitation [7–10], recent findings by Danial *et al.* [12] provide new insights into this issue. Although a higher rate of early aortic valve-related reoperations was reported in their patch group, no significant differences were observed between patched and non-patched groups on subsequent mid-term follow-ups. Therefore, the widespread adoption of patch techniques with consistent outcomes could lead to more effective management of annulus-cusp mismatch.

Ehrlich *et al.* [13] reported improved outcomes over a 25-year follow-up period using repair techniques guided by EH measurements. Maintaining an adequate EH inherently ensures sufficient CL, which is consistent with our present findings. Notably, a GH ≥18 mm has been recommended for aortic valve repair in tricuspid valves [14]. Moreover, cusp retraction, rather than prolapse, has been associated with higher rates of reoperation and recurrent regurgitation [15, 16]. This condition likely corresponds to mild or severe mismatch in our study. However, the favourable initial outcomes and absence of reoperation at 5 years post-surgery in the mild-mismatch group suggest that the annulus-cusp relationship is crucial, compared with GH alone. Successful VSRR outcomes depend on multiple technical and anatomical factors beyond the annulus-cusp relationship. The surgeon’s ability to modify annular dimensions through precise sub-annular suture placement and tension is a key technical aspect of the procedure. Furthermore, the final valve configuration involves complex interactions between EH, CL and sinotubular junction dimensions.

The use of preoperative CT measurements for predicting CL and guiding surgical planning represents a novel approach in aortic valve repair. This allows for a more precise assessment of the annulus-cusp relationship, further refining patient selection and operative strategies. The significant differences in GH and annulus diameter among the groups in this study emphasize the importance of these parameters in determining mismatch severity. Our surgical technique, involving tailoring the size of the Valsalva graft based on preoperative measurements and intraoperative findings, was found to be effective in achieving good short-term results across all groups. The low rates of moderate or severe AR at discharge, even in the severe-mismatch group, indicate that our approach can achieve satisfactory immediate outcomes. However, the divergence in mid-term results underscores the need for careful patient selection and potentially more aggressive annular reduction in cases of severe mismatch. Indeed, for certain cases of severe mismatch, valve replacement may be the more appropriate option to ensure long-term durability and reduce reoperation risk.

Collectively, our findings showed that the use of the reimplantation technique in this patient population may have contributed to the overall good outcomes, as this method provides excellent annular stabilization [4, 5]. However, in cases of severe mismatch, additional techniques, such as cusp reinforcement, more aggressive annular reduction or valve replacement, should be considered to improve long-term durability.

Despite the valuable insights provided by this study, some limitations must be acknowledged. First, this was a retrospective observational study with a relatively small sample size, which precluded us from performing multivariable analyses to adjust for potential confounders. However, the well-balanced baseline characteristics among groups and mechanical nature of the geometric relationship under study mitigate this limitation. Second, the formula used to calculate the CL assumes a uniform cusp distribution, which may not always accurately reflect the complex geometry of the aortic valve. Third, all study procedures were performed by a single, highly experienced surgeon, potentially introducing surgeon bias. Additionally, our surgical technique, including graft size selection and annular reduction strategies, evolved during the study period. Fourth, although this study focuses primarily on the annulus-cusp relationship, other critical factors, such as commissural positioning, EH management and sinotubular junction dimensions, also significantly influence surgical outcomes. Furthermore, although we prespecified our primary end-points, analyzing multiple end-points without adjustment for multiplicity increases the risk of type I error, particularly for secondary end-points. This should be considered when interpreting our results. Lastly, given the single-centre design of this study, further validation with a larger cohort is necessary to ensure generalizability of our findings across different institutions and patient populations.

## CONCLUSION

Our study demonstrated that severe annulus-cusp mismatch was associated with higher rates of AR recurrence and reoperation following aortic valve repair with VSRR. Although preoperative CT-based assessment of annulus-cusp mismatch provides valuable information for surgical planning, it should be considered alongside other critical factors, including surgical technique, commissural positioning and overall root geometry. Successful VSRR requires a comprehensive evaluation and appropriate technical execution that addresses all these components. Future studies with larger cohorts and longer follow-up periods are warranted to further validate these findings and explore potential strategies to improve outcomes in severe annulus-cusp mismatch.

## Supplementary Material

ivaf048_Supplementary_Data

## Data Availability

The data underlying this article will be shared on reasonable request to the corresponding author.

## Notes

Presented at the 38th EACTS Annual Meeting, Lisbon, Portugal, October 9–12, 2024.

## ABBREVIATIONS

AR: Aortic valve regurgitation
CI: Confidence interval
CL: Coaptation length
CPB: Cardiopulmonary bypass
CT: Computed tomography
EH: Effective height
GH: Geometric height
IQR: Interquartile range
MDCT: Multidetector-row computed tomography
VSRR: Valve-sparing aortic root replacement
