# Supplementary material for: Impact of annulus-cusp mismatch on mid-term outcomes of aortic valve repair with valve-sparing aortic root replacement
Source: Interdiscip Cardiovasc Thorac Surg. 2025 Feb 26;40(3):ivaf048. doi: 10.1093/icvts/ivaf048 (PMC11906396; doi:10.1093/icvts/ivaf048)
Supplement: ivaf048_Supplementary_Data [file ivaf048_supplementary_data.zip › Supplemental_Material.docx]

**Supplementary Data**

**Supplemental Figure 1**. Flowchart showing the selection process of the study participants.

**Supplemental Figure 2.** Cumulative incidence of moderate aortic valve regurgitation (AR) between no-mismatch and mismatch groups (A). Cumulative incidence of reoperation between no-mismatch and mismatch groups (B).
